# Metabolic contributions to neuronal deficits caused by genomic disruption of schizophrenia risk gene SETD1A

**DOI:** 10.1038/s41537-022-00326-9

**Published:** 2022-12-29

**Authors:** Zheng-Shan Chong, Zi Jian Khong, Shermaine Huiping Tay, Shi-Yan Ng

**Affiliations:** 1 Cellular Basis of Neural Diseases Laboratory, Institute of Molecular and Cell Biology, A*STAR Research Entities, Singapore, Singapore; 2 Integrative Sciences and Engineering Programme, NUS Graduate School, National University of Singapore, Singapore, Singapore; 3 National University of Singapore, Yong Loo Lin School of Medicine (Department of Physiology, Singapore, Singapore; 4 National Neuroscience Institute, Singapore, Singapore

**Keywords:** Developmental biology, Molecular neuroscience, Schizophrenia, Genetics of the nervous system

## Abstract

Regulation of neuronal metabolism during early brain development is crucial for directing synaptic plasticity and proper circuit formation. Alterations in neuronal glycolysis or mitochondrial function are associated with several neuropsychiatric disorders, including schizophrenia. Recently, loss-of-function mutations in SETD1A, a histone methyltransferase, have been linked to increased schizophrenia risk and global developmental delay. Here, we show that heterozygous disruption of SETD1A in human induced pluripotent stem cell (hiPSC)-derived neurons results in reduced neurite outgrowth and spontaneous activity, two phenotypes commonly associated with schizophrenia, as well as alterations in metabolic capacity. Furthermore, supplementing culture media with metabolic intermediates ameliorated changes in neurite outgrowth and spontaneous activity, suggesting that metabolic dysfunction contributes to neuronal phenotypes caused by SETD1A haploinsufficiency. These findings highlight a previously unknown connection between SETD1A function, metabolic regulation, and neuron development, and identifies alternative avenues for therapeutic development.

## Introduction

Neurodevelopmental disorders arising from inborn errors of metabolism underscore the importance of energy production and regulation in the developing brain^1^. Neuronal transmission is an energy-intensive activity that relies on large amounts of ATP generated by glycolysis and mitochondrial respiration^2,3^. Transient changes in metabolism are also crucial for directing synaptic plasticity and shaping neuronal circuits during development. Neurological symptoms of patients with metabolic disorders often include global developmental delay, epilepsy, cerebellar ataxia, and occasionally craniofacial dysmorphisms^4–7^. On the other hand, certain genetic variants associated with complex neurodevelopmental disorders like autism and schizophrenia have also been linked to mitochondrial dysfunction^8,9^.

Heterozygous loss-of-function mutations in SETD1A have been associated with increased risk of schizophrenia and intellectual disability^10,11^. SETD1A belongs to the SET1/COMPASS family of H3K4 histone methyltransferases, and is widely expressed in different tissues, including the developing brain^12^. Interestingly, patients with SETD1A haploinsufficiency also present with global developmental delay and craniofacial dysmorphism, with a small percentage experiencing seizures in early childhood^13,14^. Furthermore, SETD1A has been previously reported to regulate glycolysis in gastric cancer^15^ and alter neuronal network activity through the cAMP/PKA pathway, a known regulator of mitochondrial dynamics and function^16,17^. However, the effects of SETD1A deficiency on neuronal metabolism and its impact on neurodevelopment remains poorly understood.

To address this, we generated isogenic human iPSC lines harbouring heterozygous frameshift mutations in exon 7 of SETD1A and differentiated them into cortical spheroids. SETD1A^+/−^ neurons derived from spheroids showed reduced neurite outgrowth and spontaneous activity, phenotypes that were also observed in mouse models of the disorder^18,19^. In addition, we found alterations in basal glycolysis and spare respiratory capacity in SETD1A^+/−^ neurons. Supplementation of culture media with pyruvate restored neurite outgrowth and spontaneous activity in SETD1A^+/−^ neurons, suggesting that metabolic dysfunction contributes to neuronal deficits caused by SETD1A disruption. Our findings indicate that SETD1A plays a role in regulating neuronal metabolism during development and identifies metabolism as a potential therapeutic target for treating developmental disorders caused by SETD1A haploinsufficiency.

## Methods

### Cell culture and genome editing

hiPSCs derived from BJ fibroblasts (ATCC no. CRL-2522; BJ-iPSCs) and H7 human embryonic stem cells (WiCell no. WAe007-A) were cultured feeder-free on Matrigel-coated dishes in StemMACS iPS-Brew XF (Miltenyi Biotec). Routine passaging was performed every 6–7 days using ReLeSR (Stemcell Technologies). Guide RNA were designed using the gRNA tool on Benchling. Genome editing was performed as previously described^20^, using gRNA targeting exon 7 of SETD1A, and mutant BJ-iPSC or H7 clones verified by Sanger sequencing. gRNA and sequencing primers used are listed in Supplementary Table S1. Mutant lines were screened for chromosome abnormalities using the KaryoStat Assay (ThermoFisher).

### Cortical spheroid differentiation

Cortical spheroid differentiation was performed as previously described^21^, with some modifications. Pluripotent stem cell colonies were dissociated using Accutase (Stemcell Technologies) and seeded at 1.5 × 10^4^ cells per well on ultra-low attachment U-bottom plates. For neural induction, 0.5 μM LDN193189 (Sigma-Aldrich) was used in place of dorsomorphin. N2B27 media (50% DMEM/F12, 50% Neuro medium, 1% L-Glutamax, 1% MEM Non-Essential Amino Acids supplemented with 1% N2 supplement, and 2% B27 without Vitamin A supplement) was used throughout the cortical spheroid differentiation with the addition of relevant growth factors.

### SDS-PAGE and western blot

Spheroids and iPSCs were lysed in RIPA lysis buffer (ThermoFisher) with protease inhibitors. Protein lysates were resolved using 4–20% precast gels in Tris-Glycine-SDS buffer. Proteins were transferred to a nitrocellulose membrane using the Trans-Blot Turbo system (Bio-Rad) and blocked with 5% milk in PBS with 0.1% Tween-20. Membranes were incubated in primary antibodies overnight at 4 °C, washed and incubated in the corresponding HRP-conjugated secondary antibodies for 1 h at room temperature. Proteins were detected using SuperSignal West Femto Maximum Sensitivity Substrate (ThermoFisher) and images acquired using the ChemiDoc Gel Imaging system (Bio-Rad). Primary and secondary antibodies and dilutions used are listed in Supplementary Table S2.

### RNA isolation and q-RT-PCR

Cells were harvested in TRIzol (Invitrogen) for RNA extraction following manufacturer’s instructions. Purified RNA was converted to cDNA using the High-Capacity cDNA Reverse Transcription kit (ThermoFisher), and qPCR performed on the QuantStudio 5 Real-Time PCR System using PowerUp SYBR Green Master Mix (Applied Biosystems). Gene expression levels were normalized to β-Actin expression. Relative expression was calculated using the ΔΔCt method. All primer sequences used are listed in Supplementary Table S3.

### Cryosectioning and Immunofluorescence

Spheroids were fixed in 4% paraformaldehyde (PFA) and sectioned at 10 μm thickness. 2D neuronal cultures were similarly fixed in 4% PFA before staining. Neuronal cultures and spheroid sections were permeabilised, blocked and then stained with primary antibodies overnight at 4 °C. Slides/plates were incubated with the corresponding fluorophore-conjugated secondary antibodies and DAPI for 1.5 h at room temperature before imaging. All primary antibodies and dilutions used are listed in Supplementary Table S2. Image acquisition for spheroid sections were performed on a Ti-E microscope (Nikon) and analysed with ImageJ (Fiji). To avoid bias during image analysis, labels were removed during quantification and images analysed in a randomized order.

### Neurite outgrowth assay

D16 spheroids were plated on Matrigel-coated plates in N2B27 media without additional growth factors. Brightfield images of spheroids were taken and resulting neurite measured after 48 h in static culture. Images from wells were acquired following a randomized order and labels removed before analysis. Neurite outgrowth was quantified with ImageJ (Fiji) by defining the boundaries of the main spheroid body and measuring the length to the furthest neurite extension in perpendicular directions. At least 4 measurements were taken and averaged per spheroid. Genotypes/treatment information were matched back to samples after quantification. Plated spheroids were subsequently fixed with 4% PFA and stained for Nestin and DCX (refer to Supplementary Table S2 for list of antibodies used in this study).

### Calcium imaging

Live imaging was performed on a Ti2-E fluorescence microscope (Nikon) equipped with temperature and CO_2_ control. For calcium imaging, Calcium 6 Assay dye (Molecular Devices, R8190) was prepared according to manufacturer’s instructions and incubated with cells for 2 h at 37 °C before imaging. Imaging was performed at 488 nm excitation, with frames taken every 500 ms for 2 min after a 30 s delay for baseline fluorescence to stabilise. Data analysis of calcium imaging was performed using ImageJ (Fiji) and custom R scripts for spike peak detection. ROIs representing active neurons were manually selected after taking the difference between maximum fluorescence intensity and fluorescence at T_0_ (first frame). Fluorescence intensity values were recorded and change in fluorescence was calculated as follows: ΔF/F = (F − F_0_))/F_0_, in which F_0_ refers to mean fluorescence at T_0_. Calcium spike peaks were defined based on a semi-automated workflow using the peakPick package in R^22^ to output peak counts along with annotated plots showing automatically detected peaks. Peaks identified from each recording were manually screened (with genotype labels removed) to ensure accurate peak counts.

### Seahorse and lactate release assays

Extracellular acidification rate (ECAR) and oxygen consumption rate (OCR) were measured using an XFe96 Seahorse Biosciences Extracellular Flux Analyzer (Agilent Technologies). Cortical spheroids were dissociated and plated onto Matrigel-coated Seahorse 96-well plate at 125,000 neurons per well. Neuronal cultures were treated with 2 μM cytosine arabinoside (Ara-C, Sigma-Aldrich) for 48 h to eliminate any remaining neural progenitors and enrich for mature neurons. To measure glycolytic rate and maximum glycolytic capacity, we recorded neuronal ECAR after 1 h of glucose deprivation and sequential treatment with 10 mM glucose, 2 μM oligomycin, and 50 mM 2-deoxy-glucose (2-DG). Respiratory capacity was similarly determined by measuring OCR during sequential treatment of cells with 2 μM oligomycin, 1 μM FCCP, and 0.5 μM rotenone/Antimycin A, in the presence of 10 mM glucose. Each treatment lasted 18 min with ECAR and OCR measurements taken every 6 min. Cell number per well was determined post assay using Hoechst 33342 staining (ThermoFisher) for normalisation of ECAR/OCR values. Lactate release was measured using 25 μl of cortical neuron conditioned media after 2 h, using the L-lactate Assay Kit (Abcam) as per manufacturer’s instructions.

### Metabolite supplementation

For neurite outgrowth assays, spheroids were grown in N2B27 media supplemented with 1 mM sodium pyruvate (Gibco), 1 mM sodium L-lactate (Sigma-Aldrich) or 0.5 mM D-Glucose (Sigma-Aldrich) for 48 h on Matrigel-coated plates. For calcium imaging, neuronal cultures were grown in cortical maturation media containing N2B27 with 20 ng/μl BDNF and 20 ng/μl NT-3 and supplemented with 1 mM sodium pyruvate (Gibco) or 1 mM sodium L-lactate (Sigma-Aldrich) for two weeks. Media was changed every alternate day.

## Results

### SETD1A^+/−^ cortical spheroids exhibit mild growth deficits

**Fig. 1:**
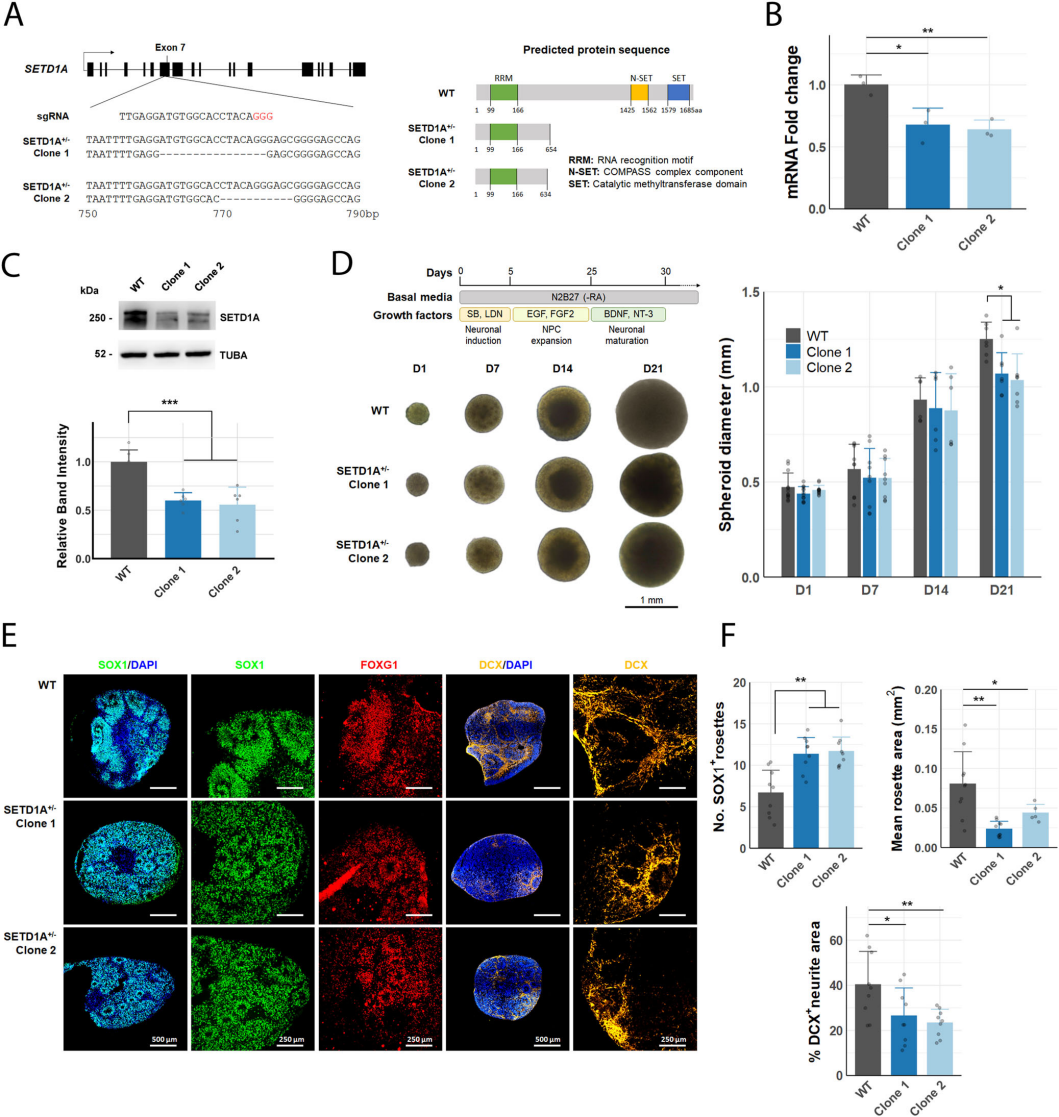
Heterozygous disruption of SETD1A leads to neurodevelopmental phenotypes in cortical spheroids. **A** Frameshift mutations induced in SETD1A^+/−^ hiPSC lines and respective predicted protein sequences. **B** Relative expression of SETD1A transcript in WT and SETD1A^+/−^ iPSCs (*n* = 3 wells from 3 independent passages). **C** Representative bands from western blot of SETD1A in WT and SETD1A^+/−^ iPSCs and relative quantification of band intensity (*n* = 6 wells from 2 independent passages). **D** Cortical spheroid differentiation protocol and respective brightfield images of spheroids during the first three weeks of differentiation. (left) Quantification of WT and SETD1A^+/−^ spheroid diameter. (*n* = 9 spheroids per timepoint per line from 3 independent differentiations). **E** Representative IF images of spheroid sections at D21. **F** Quantification of (top left) SOX1^+^ rosette number, (top right) mean rosette area, (bottom) DCX^+^ area as a percentage of DAPI^+^ area (*n* = 9 spheroids per line from 3 independent differentiations) Data represented are mean ± sd. For all comparisons, one-way ANOVA with Dunnett’s multiple comparisons test was performed. *p < 0.05; **p < 0.01.

To investigate the effect of SETD1A haploinsufficiency in human neurons, we generated SETD1A^+/−^ hiPSC lines using transient expression of CRISPR/Cas-9 nuclease and a gRNA targeting exon 7 (Fig. 1A). We chose to target exon 7 as it is a site where frameshift mutations have been identified in multiple patients^13^. Sanger sequencing of the edited locus revealed heterozygous frameshift deletions in two mutant lines obtained (SETD1A^+/−^ Clone 1, Clone 2), which were not present in the parental cell line (Supp. Fig. S1A). Both deletions are predicted to result in an early stop codon before the SET methyltransferase domain, which should result in a truncated, non-functional protein (Fig. 1A).

Accordingly, we observed a reduction in SETD1A expression at transcript and protein levels in SETD1A^+/−^ iPSCs compared to WT iPSCs (Fig. 1B, C). Of note, we did not observe any homozygous mutations or complete knockouts in any of the 21 clones sequenced (data not shown), consistent with previous reports that SETD1A is essential for cell survival^23^. Although SETD1A knockdown has been shown to affect genomic stability and reduce proliferation rates in cancer cells^24,25^, we found no chromosomal abnormalities or proliferation defects in our SETD1A^+/−^ iPSC lines (Supp. Fig. S1B, C). Expression of pluripotency markers NANOG and OCT4/POU5F1 in SETD1A^+/−^ iPSCs was also comparable to that in WT iPSCs (Supp. Fig. S1D). As CRISPR/Cas-9 is known to have some off-target activity, we also checked possible gRNA off-target sites within coding sequences in the genome and did not find any mutations at these sites (Supp. Fig. S1E).

We next differentiated WT and SETD1A^+/−^ iPSC lines into cortical spheroids using a previously published protocol^21^ and found that SETD1A^+/−^ cortical spheroids were consistently smaller at Day 21 post-induction (Fig. 1D). Cortical spheroids have been shown to produce laminated cerebral cortex-like structures containing neural progenitor cells (NPCs), electrically active neurons, and astrocytes^21,26^, thus serving as a useful model for early neurodevelopment. Immunofluorescence (IF) staining of cortical spheroid sections at Day 21 showed that SETD1A^+/−^ spheroids tended to have smaller but more numerous FOXG1^+^ and SOX1^+^ neural rosettes, as well as a reduction in DCX^+^ neurite area (Fig. 1E, F). The number of SOX1^+^ neural rosettes was counted manually by identifying groups of SOX1^+^ cells surrounding a central lumen (Supp. Fig. S2A), and all visible rosettes within each spheroid were counted, for 5 spheroids per line. DCX^+^ neurite area was determined using a standardised threshold in ImageJ. Genotype labels of images were removed and images quantified in a randomised order to reduce bias. To investigate the efficiency of differentiation of NPCs and neurons, cortical spheroids were dissociated at D18 and the resulting cells were cultured as a monolayer on Matrigel-coated plates to facilitate counting of individual cells. Immunofluorescent staining for the SOX2 was used to identify NPCs whilst DCX was used as a marker of immature neurons (Supp. Fig. 2B). Number of SOX2^+^ NPCs and DCX^+^ neurons were subsequently quantified using the Columbus Image Data Storage and Analysis System (Perkin Elmer). All three lines produced similar numbers of SOX2^+^ NPCs and DCX^+^ neurons (Supp. Fig. S2B), indicating that smaller spheroid sizes were likely due to changes in volume of neural processes rather than a reduction in absolute numbers of DCX^+^ neurons.

### Neurite outgrowth and spontaneous activity are reduced in SETD1A^+/−^ neurons

**Fig. 2:**
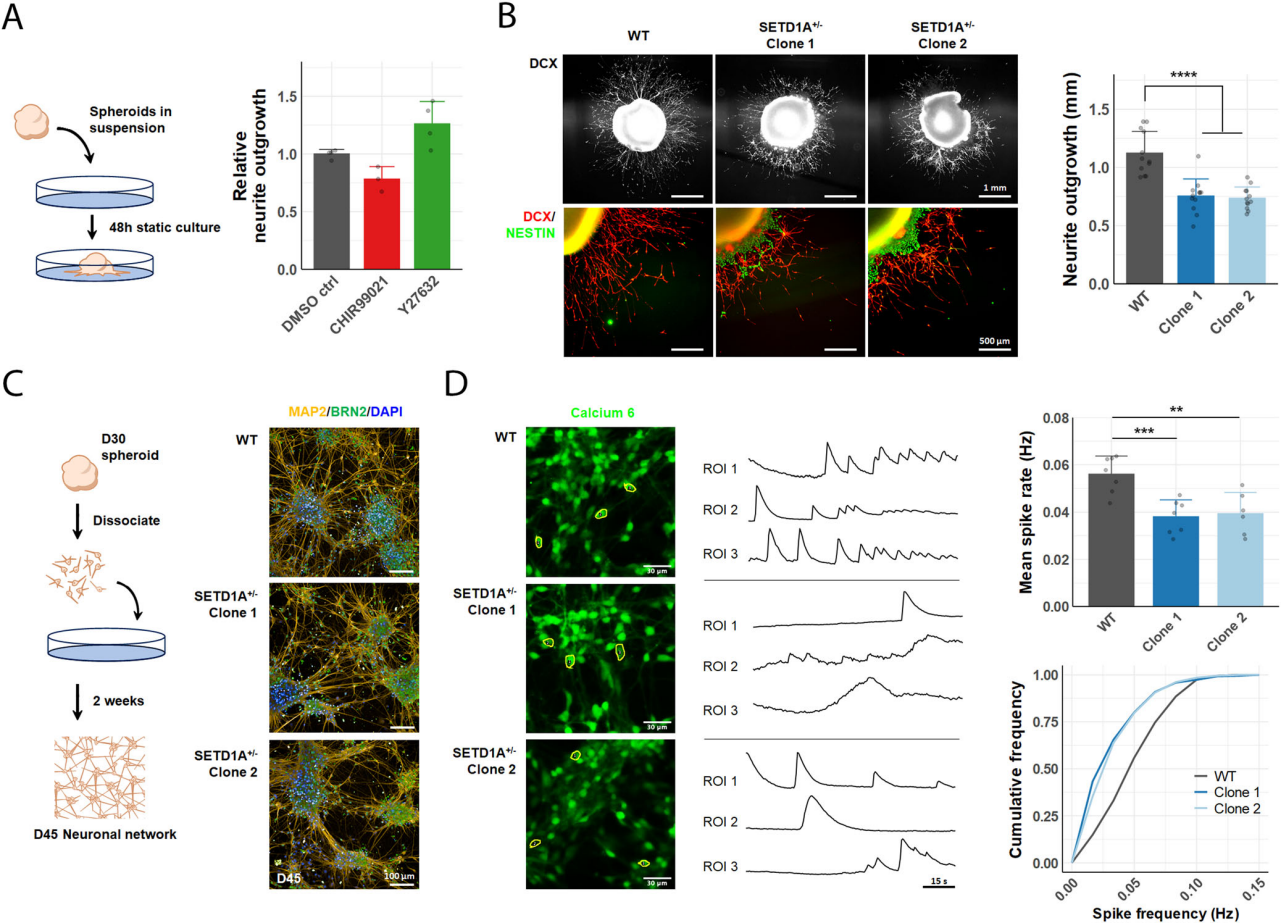
SETD1A deficient neurons recapitulate schizophrenia-associated changes in neurite outgrowth and spontaneous activity. **A** Neurite outgrowth assay and response to known inhibitors (CHIR99021) and enhancers (Y27632) of neurite outgrowth (*n* = 4 wells per condition. **B** Representative IF images of neurite outgrowth in WT and SETD1A^+/−^ spheroids after 48 h of static culture. Quantification of neurite outgrowth in WT and SETD1A^+/−^ spheroids (*n* = 12 spheroids from 3 independent differentiations). **C** Dissociation of cortical spheroids at D30 and formation of 2D neuronal networks for calcium imaging. Representative IF images of BRN2 + /MAP2 + cortical neurons at D45 in WT neurons. **D** Representative images of WT and SETD1A^+/−^ neurons after 2 h incubation with Calcium 6 dye. Representative calcium imaging traces showing ΔF/F_0_ over time for neurons outlined in yellow. (top right) Mean spike rate of active neurons within a neuronal network for WT and SETD1A^+/−^ cells (*n* = 7 wells (WT, SETD1A^+/−^ Clone 1), 6 wells (SETD1A^+/−^ clone 2) from 3 independent differentiations). (bottom right) Cumulative fraction of neurons by spike frequency (*n* = 302 (WT), 273 (SETD1A^+/−^ Clone 1), 364 (SETD1A^+/−^ Clone 2) neurons recorded, from 3 independent differentiations). Data represented are mean ± sd. For all comparisons, one-way ANOVA with Dunnett’s multiple comparisons test was performed *p < 0.05; **p < 0.01; ***p < 0.001; ****p < 0.0001.

To further investigate neurite outgrowth in SETD1A^+/−^ cortical spheroids, we optimised an assay to measure neurite extension that involved plating spheroids onto a Matrigel-coated plate and incubating them in N2B27 at 37 °C for 48 h, without shaking (Fig. 2A). Following 48 h of static culture, we observed outgrowth of DCX^+^ neurites from the main spheroid body in in all directions along the plate (Supp. Fig. S2C). We validated this assay by transiently treating the plated spheroids with 1 μM of either CHIR99021 or Y-27632 for 48 h, and measuring resulting neurite outgrowth (Fig. 2A, Supp. Fig. S2C). CHIR99021 is a GSK-3β inhibitor that has been shown to inhibit neurite outgrowth in NGN2-induced neurons^27^, while Y-27632 inhibits Rho‑associated coiled‑coil containing protein kinase (ROCK) and has been previously shown to enhance neurite outgrowth in PC12 cells^28^.

Using this assay, we found that DCX^+^ neurite outgrowth was indeed reduced in SETD1A^+/−^ spheroids as compared to WT (Fig. 2B). In addition, we observed migration of Nestin^+^ neural progenitors out of the main spheroid body more frequently in SETD1A^+/−^ spheroids, suggesting possible defects in NPC migration (Fig. 2B).

As previous studies have reported conflicting alterations in spontaneous firing in SETD1A^+/−^ neurons^17,18^, we decided to investigate neuronal activity in our SETD1A^+/−^ lines. To facilitate calcium imaging, we dissociated cortical spheroids at Day 30 and cultured them as a monolayer for 2 weeks to allow the formation of 2D neuronal networks (Fig. 2C). Dissociated cell cultures were treated with 2 μM Ara-C for 48 h to eliminate non-neuronal cells and enrich for mature neurons (Supp. Fig. S3A). Furthermore, the expression levels of neural progenitor (SOX1/FOXG1) and neuronal (TUJ1/BRN2) markers as measured by qRT-PCR were similar between WT and SETD1A^+/−^ cultures, suggesting a similar ratio of NPCs to neurons in all three cell lines (Supp. Fig. S3B).

Calcium imaging was performed on D45 neuronal cultures as immunofluorescence staining of WT neurons showed co-expression of mature neuronal marker MAP2 and presynaptic marker SV2, indicating that they should be electrically active (Supp. Fig. S3C). In brief, neuronal cultures were incubated with Calcium 6 dye for 2 h at 37 °C and recorded for 2 min after a 30 s delay for baseline fluorescence to stabilise. Active neurons were identified and calcium spikes identified using the peakPick package in R^22^ (Supp. Fig. S3D). Spontaneous calcium transients were observed in neurons derived from all three lines, although the frequency of firing was significantly reduced in SETD1A^+/−^ neurons (Fig. 2D). Cumulative frequency curves showed a leftward shift for SETD1A^+/−^ lines, suggesting that reductions in mean spike rate was due to a uniform decrease in firing frequency across the whole population rather than changes in differentially active subpopulations (Fig. 2D). Our data are consistent with findings from mice models which showed reduced neurite complexity and impaired synaptic transmission in the frontal cortex of SETD1A^+/−^ mice^18,19^.

### SETD1A^+/−^ neurons have altered metabolic capacities

**Fig. 3:**
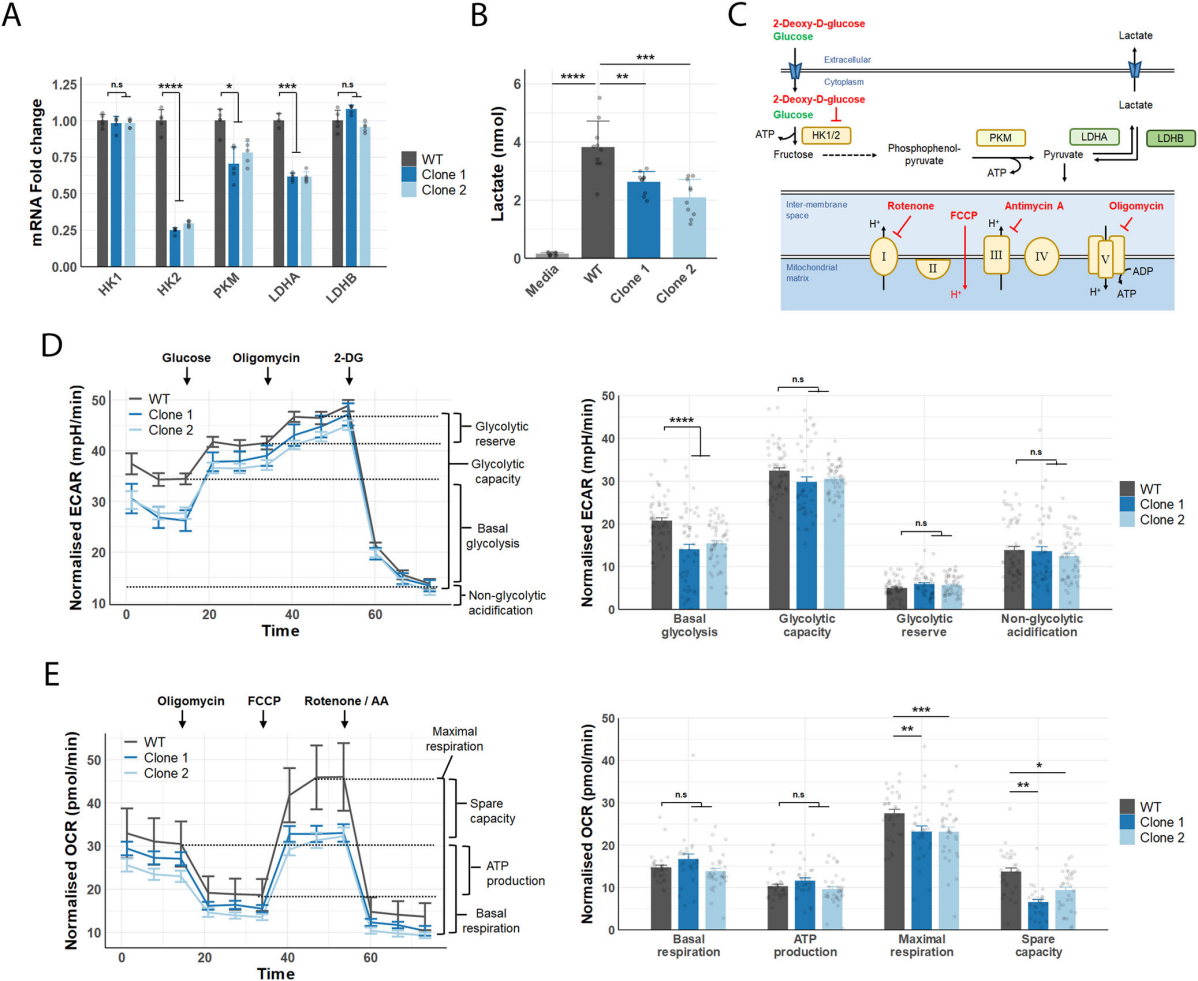
Altered basal glycolysis and spare respiratory capacity in SETD1A deficient neurons. **A** Relative expression of glycolytic enzymes in D30 cortical spheroids (*n* = 3 wells per line, from 3 independent differentiations). **B** Lactate levels in media obtained from D34 neuronal cultures (*n* = 10 wells per line from 2 independent differentiations). **C** Diagram showing glycolysis pathway and mitochondrial respiratory complex, highlighting the effects of small molecule inhibitors used in Seahorse assays to measure glycolytic and mitochondrial stress. **D** Results of glycolytic stress assay in WT and SETD1A^+/−^ neurons at D34. (left) Seahorse curves showing basal glycolysis and responses to 10 mM glucose, 2 μM oligomycin and 50 mM 2-DG over time (mins). Extracellular acidification rate (ECAR) was normalised to nuclei number post assay. (right) Quantification of glycolytic function from the same experiments (error bars = sem, *n* = 55 well (WT), 52 wells (SETD1A^+/−^ Clone 1), 53 wells (SETD1A^+/−^ Clone 2) from 3 independent differentiations). **E** Results of mitochondrial stress assay in WT and SETD1A^+/−^ neurons at D34. (left) Seahorse curves showing basal respiration and responses to 2 μM oligomycin, 1 μM FCCP, and 0.5 μM rotenone/antimycin-A over time (mins). Oxygen consumption rate (OCR) was normalised to nuclei number post assay. (right) Quantification of respiratory function from the same experiments (error bars = sem, *n* = 32 wells (WT), 27 wells (SETD1A^+/−^ Clone 1), 33 wells (SETD1A^+/−^ Clone 2) from 3 independent differentiations). Data represented are mean ± sd unless otherwise indicated. For all comparisons, one-way ANOVA with Dunnett’s multiple comparisons test was performed *p < 0.05; **p < 0.01; ***p < 0.001; ****p < 0.0001.

SETD1A overexpression has been shown to regulate the expression of key glycolytic genes in gastric cancer^15^. Therefore, we hypothesised that SETD1A loss-of-function might cause changes in glycolytic gene expression and therefore affect neuronal metabolism. We observed a significant reduction in transcript levels of several glycolytic enzymes including HK2, PKM, and LDHA in SETD1A^+/−^ cortical spheroids (Fig. 3A), as well as a significant reduction in lactate release in the supernatant of SETD1A^+/−^ neuronal cultures (Fig. 3B). Lactate is a by-product of glycolysis that is commonly used as a proxy to measure glycolytic rate; our results suggest that disruption of SETD1A might affect glycolytic function.

To further investigate the metabolic effects of SETD1A disruption, we used Seahorse metabolic flux assays to assess glycolytic capacity and mitochondrial function in WT and SETD1A^+/−^ neurons. These assays measure the response of live cells to various metabolic substrates and inhibitors (Fig. 3C), using extracellular acidification and oxygen consumption as a readout for glycolysis and oxidative phosphorylation, respectively. As different cell types have distinct metabolic profiles, we treated dissociated cortical spheroids with 2 μM Ara-C for 48 h to eliminate non-neuronal cell types. Results from qRT-PCR of NPC and neuronal markers suggested that WT and SETD1A^+/−^ neuronal cultures had similar cell-type compositions. (Supp. Fig. S4A, B). Additionally, immunostaining of WT and SETD1A^+/−^ neuronal cultures showed comparable levels of NPCs with the absence of astrocytes within the culture (Supp. Fig. 5A, B).

Interestingly, we found that SETD1A^+/−^ neurons had lower basal glycolysis rates after 1 h of glucose deprivation, although glycolysis under high-glucose conditions and maximal glycolytic capacity were not affected (Fig. 3D). Since oxidative phosphorylation is sometimes enhanced to compensate for reduced ATP when glycolysis is impaired^29^, we also measured mitochondrial function in SETD1A^+/−^ neurons. Surprisingly, we did not observe any differences in basal respiration and ATP production between WT and SETD1A^+/−^ neurons. However, SETD1A^+/−^ neurons showed lower spare and maximal respiratory capacity (Fig. 3E). This suggests that whilst SETD1A^+/−^ neurons are metabolically normal under ideal conditions, they might be more susceptible to metabolic stress, for instance during times of low glucose supply or increased ATP demand.

Given the possibility that such metabolic alteration could be a defect exclusive to BJ-iPS lines, we generated a separate set of H7 SETD1A^+/−^ lines using the same CRISPR/Cas-9 approach as those performed on the BJ-iPS line. (Supp. Fig. 6A, B). Glycolytic capacity and mitochondrial function were measured for the H7 SETD1A^+/−^ lines accordingly. These H7 SETD1A^+/−^ neurons exhibited comparable reduction in both basal glycolysis rates and maximal respiratory capacity that were observed in the BJ-iPS lines (Supp. Fig. 6C, D). These results further highlight that such metabolic alterations are not exclusive to BJ-iPS lines but a consequence of SETD1A haploinsufficiency.

We next asked whether reduction in glycolytic capacity was a primary or secondary consequence of SETD1A knockdown using transient siRNA knockdown of SETD1A. Transfection of siRNA into D34 WT neurons from BJ or H7 lines resulted in a robust knockdown of SETD1A 72 h post transfection, along with significant reductions in LDHA and LDHB transcript expression (Supp. Fig. S4C). This indicates that SETD1A directly affects transcription of some glycolytic genes in neurons. SETD1A is histone methyltransferase known to methylate H3K4, a histone mark associated with gene activation^30,31^. Notably, previous ChIP-seq studies of SETD1A binding in prefrontal mouse cortex^19^ showed possible binding of SETD1A to the promoter regions of several glycolytic enzymes including HK1, PKM and LDHA (Supp. Fig. S4D), suggesting a possible mechanism for the effect of SETD1A on neuro-metabolism.

### Pyruvate supplementation ameliorates neuronal deficits caused by SETD1A disruption

**Fig. 4:**
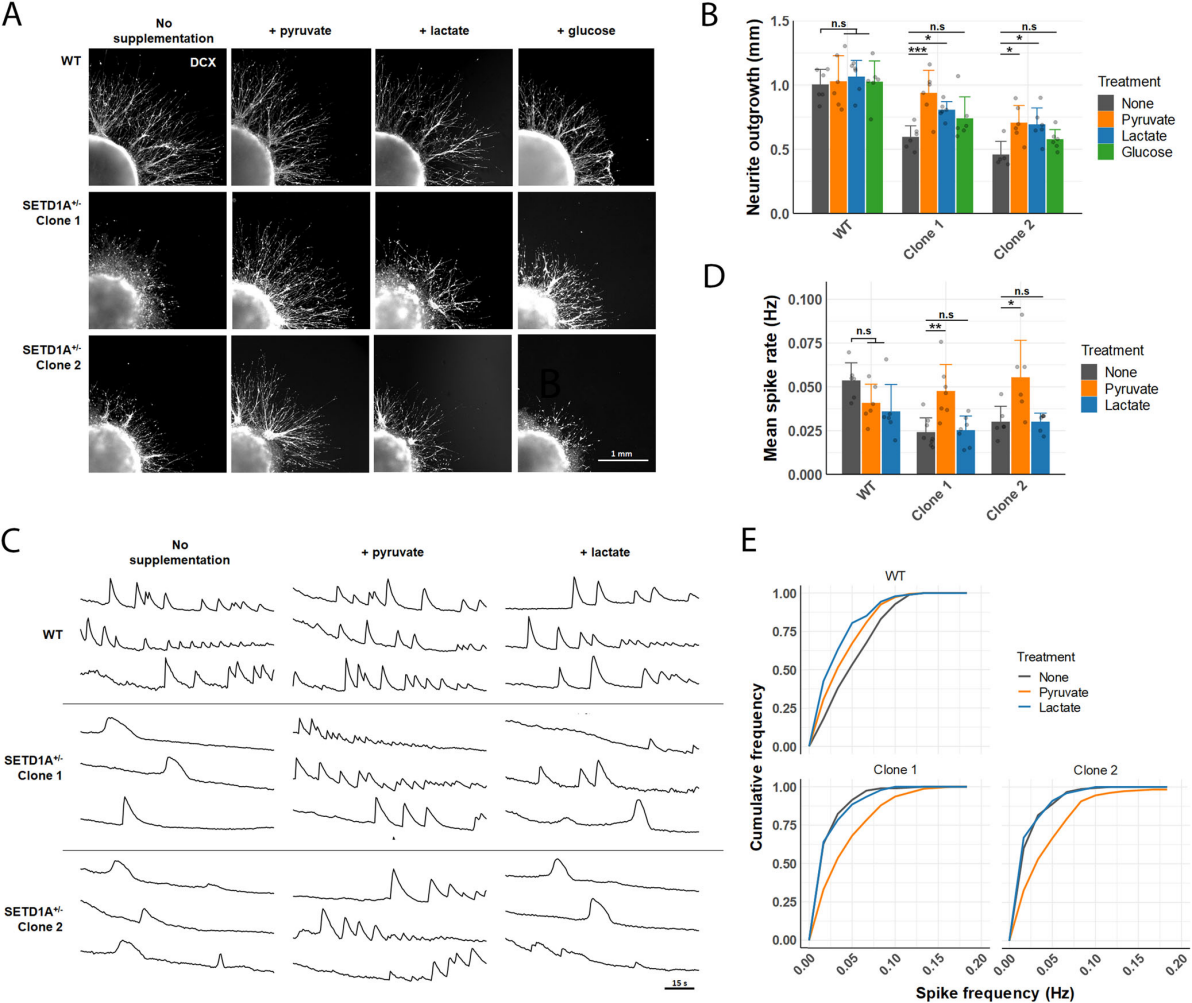
Pyruvate supplementation ameliorates neuronal phenotypes caused by SETD1A haploinsufficiency. **A** Representative IF images of neurite outgrowth from cortical spheroids after 48 h of static culture in media supplemented with either 1 mM pyruvate, 1 mM lactate, or 0.5 mM glucose. **B** Quantification of neurite outgrowth in response to metabolite supplementation (*n* = 6 spheroids per line per treatment, from 3 independent differentiations. **C** Representative calcium imaging traces of ΔF/F_0_ over time in active neurons after 2 weeks of metabolite supplementation. **D** Mean spike rate of active neurons within a neuronal network after 2 weeks of metabolite supplementation (*n* = 6 wells per line per treatment, from 3 independent differentiations). **E** Cumulative fraction of neurons by spike frequency (*n* = 213 (WT/no treatment), 202 (WT/pyruvate), 208 (WT/lactate); 217 (Clone 1/no treatment), 162 (Clone 1/pyruvate), 155 (Clone 1/lactate); 153 (Clone 2/no treatment), 183 (Clone 2/pyruvate), 100 (Clone 2/lactate) neurons recorded, from 3 independent differentiations). Data represented are mean ± sd. For all comparisons, two-way ANOVA with Sidak’s multiple comparisons test was performed. *p < 0.05; **p < 0.01; ***p < 0.001; ****p < 0.0001.

Given that metabolic regulation is critical for proper neurodevelopment, and that SETD1A disruption leads to altered metabolic capacities in neurons, we wondered if increased availability of metabolic substrates could act to bolster energy production and mitigate neuronal defects caused by SETD1A haploinsufficiency. We tested this by performing neurite outgrowth assays in media supplemented with equicaloric concentrations of pyruvate, lactate, or glucose (Fig. 4A). Lactate and pyruvate can be taken up by neurons and have been previously shown to increase neuronal ATP levels^32^. Without supplementation, SETD1A^+/−^ spheroids showed reduced neurite extension after 48 h, as previously observed. However, neurite outgrowth was significantly improved in SETD1A^+/−^ spheroids treated with pyruvate or lactate (Fig. 4B). Surprisingly, addition of glucose did not significantly increase neurite extension in SETD1A^+/−^ spheroids, suggesting that reduced basal glycolysis might be a factor limiting neurite outgrowth.

We further hypothesised that an increase in neurite outgrowth might lead to more synaptogenesis and higher connectivity within neural networks, thereby increasing the frequency of spontaneous activity. To investigate this, WT and SETD1A^+/−^ neurons were cultured in media supplemented with pyruvate or lactate for two weeks, to allow for neurite outgrowth and synaptogenesis to occur before the level of spontaneous activity was assessed. Again, we observed a significantly lower frequency of calcium transients in SETD1A^+/−^ vs WT neurons without supplementation (Fig. 4C–E). Addition of pyruvate increased mean spike frequencies in SETD1A^+/−^ neurons to levels similar to that in WT neurons, whilst lactate supplementation had no effect on neuronal firing (Fig. 4C–E). It should be noted that lactate is known to have non-metabolic effects on neuronal activity, including the suppression of spontaneous calcium transients in cortical neurons^33,34^, which might explain its apparent lack of efficacy in modulating firing frequency in SETD1A^+/−^ neurons. Altogether, these results indicate that metabolic dysregulation contributes to neuronal phenotypes caused by SETD1A deficiency, and that augmenting metabolic function could be a potential therapeutic strategy for this neurodevelopmental condition.

## Discussion

In this study, we generated a human iPSC model of SETD1A haploinsufficiency that recapitulates several neuronal phenotypes reported in previous studies on SETD1A^+/−^ mice, namely impaired neurite outgrowth and reduced spontaneous activity^18,19^. Importantly, these two phenotypes have also been observed in other animal and iPSC models of schizophrenia^35–37^. In addition, we demonstrate that SETD1A loss-of-function also leads to metabolic dysregulation and that providing SETD1A^+/−^ neurons with alternative metabolic substrates like pyruvate and lactate is sufficient to rescue neuronal defects.

Our data suggests a conserved role for SETD1A in mammalian brain development, and mild differences in spheroid size between WT and SETD1A^+/−^ lines are consistent with clinical observations that patients with SETD1A-mediated developmental delay did not present with severe microcephaly or major structural brain defects^13^. Furthermore, we did not find significant changes in differentiation efficiency of SETD1A^+/−^ iPSCs to SOX2^+^ NPCs and DCX^+^ neurons, supporting the view that deficits in neural progenitor proliferation or differentiation are unlikely to be major drivers of neurodevelopmental symptoms caused by SETD1A haploinsufficiency^13^.

However, we noted more diffuse patterns of SOX1^+^ NPCs in SETD1A^+/−^ spheroid sections and an increased propensity for SETD1A^+/−^ NPCs to migrate out of cortical spheroids when grown in static culture, suggesting possible abnormalities in neural progenitor migration or adhesion. Of note, changes in cell-cell adhesion have been associated with other genetic risk factors for schizophrenia, such as in DISC1 and NRXN1 mutations^36,38–40^. However, further studies are needed to elucidate the role of SETD1A in neuronal migration.

On the effect of SETD1A haploinsufficiency in neurons, a study by Wang and colleagues previously reported an increase in dendritic complexity and network activity in NGN2-induced neurons^17^, in contrast to was observed in this study. Although both studies used iPSC-derived models, a key difference is that NGN2-induction bypasses the neural progenitor stage and converts iPSCs directly to neurons, whilst cortical spheroid differentiation produces intermediate SOX1^+^/FOXG1^+^ progenitor cells which eventually differentiate into neurons. It is therefore possible that the reduced neurite outgrowth and spontaneous activity observed in this study represents an early-stage effect of SETD1A haploinsufficiency as compared to what was reported by Wang et al. Interestingly, our findings are in line with observations from animal studies where heterozygous loss of SETD1A led to attenuated firing in cortical neurons and a decrease in dendritic complexity in adult mice^18,19^. This suggests that the role of SETD1A in neurodevelopment might not be that straightforward and that future studies are needed to investigate the downstream effects of SETD1A haploinsufficiency over time.

The metabolic phenotypes we observed in SETD1A^+/−^ neurons represent a previously unknown role for SETD1A in regulating neuronal metabolism. Although SETD1A overexpression in gastric cancer is known to promote glycolysis by increasing activating H3K4 methylation marks at HK2 and PFK2 genomic loci^15^, not much is known about the effects of partial SETD1A loss-of-function in the context of neuron development. Our results suggest that SETD1A haploinsufficiency does not affect glycolytic capacity and basal respiration rates under glucose- and oxygen-rich environments but causes reduced glycolysis after glucose deprivation and lower spare respiratory capacity. Perinatal hypoxia is also a risk factor for psychotic illness in later life^41^, so it would be interesting to know if metabolic dysregulation predisposes SETD1A^+/−^ neurons to metabolic stress, as that might explain incomplete penetrance of psychotic illness in individuals with SETD1A mutations.

In gastric cancer, SETD1A is typically overexpressed and has been shown to increase methylation of H3K4, an activating epigenetic mark, at HK2 and PFK2 promoters, thereby enhancing HIF1α recruitment needed to promote transcription of glycolytic genes^15^. We hypothesise that SETD1A might function similarly in neurons, by binding to and methylating promoters of glycolytic genes. Conversely, SETD1A haploinsufficiency would reduce H3K4 methylation at these sites, thereby downregulating glycolytic gene expression. In line with this hypothesis, ChIP-seq data from prefrontal cortex of WT mice^19^ showed SETD1A binding upstream of several glycolytic genes including HK1, PKM and LDHA (Supp. Fig. S4D).

Glycolysis and mitochondrial respiration are not only crucial for energy production but also for the biosynthesis of important macromolecules fuelling neuronal development and function^42–44^. In particular, glycolysis promotes neurite outgrowth by increasing the availability of lipid biosynthesis precursors like acetyl-CoA^45^. Pyruvate is an intermediate product of glycolysis which acts as a substrate for both mitochondrial respiration and acetyl-CoA synthesis. We found that supplementation with pyruvate, but not glucose, promoted neurite outgrowth and spontaneous activity in SETD1A^+/−^ neurons, suggesting that glycolysis may be a rate limiting step underlying reduced neurite outgrowth in SETD1A-deficient neurons.

Pyruvate can also be derived from lactate, which may be provided exogenously through the astrocyte-neuron lactate shuttle^42^. In SETD1A^+/−^ neurons, we observed an increase in neurite outgrowth but not spontaneous activity in response to lactate supplementation. This could be explained by non-metabolic effects of lactate, which is known to induce a reversible decrease in cortical neuron firing through the HCA1 receptor, an effect not seen with similar concentrations of pyruvate or glucose^33^. As astrocytes provide metabolic support for neurons mainly in the form of lactate, this has implications on the extent to which metabolic support from glial cells can compensate for deficits in neuronal glycolysis in SETD1A^+/−^ cells. Further studies are therefore needed to understand role of SETD1A in astrocytes and to identify any non-cell-autonomous effects of SETD1A deficiency on neuronal physiology.

Interestingly, lactate levels are elevated in the brains of schizophrenic individuals and negatively correlate with cognitive function^46,47^. This contrasts with our observation that SETD1A^+/−^ neurons exhibited reduced levels of lactate release. However, our model is limited in the complexity of cell types it can generate—cortical spheroids do not fully recapitulate the in vivo environment of the developing brain, although they provide more complexity than neuronal monocultures^48^. As SETD1A loss-of-function mutations are associated with increased schizophrenia risk, our model may also reflect a state of increased vulnerability rather than active disease pathophysiology. This therefore raises a question of whether elevated lactate levels are a result of, or a compensatory response to disease state in schizophrenia.

Nonetheless, our results demonstrate the utility of hiPSC-based models for studying the role of SETD1A in early brain development and identifies a previously unreported metabolic phenotype in neurons caused by SETD1A disruption. Our data also provides some preliminary evidence for the use of nutritional supplementation to reverse deficits in neurite outgrowth and activity in SETD1A^+/−^ neurons, which could serve as a foundation for further exploration of metabolic pathways as a therapeutic target for SETD1A-mediated developmental disorders.

## Supplementary information

Supplemental Figures and Tables
