# Supplementary material for: Metabolic contributions to neuronal deficits caused by genomic disruption of schizophrenia risk gene SETD1A
Source: Schizophrenia (Heidelb). 2022 Dec 29;8(1):115. doi: 10.1038/s41537-022-00326-9 (PMC9800576; doi:10.1038/s41537-022-00326-9)
Supplement: Supplementary file 1 — Supplemental Figures and Tables [file 41537_2022_326_MOESM1_ESM.doc]

**Supplementary Material**

**Supplementary Methods**

*siRNA knockdown in cortical neurons*

Cortical spheroids were dissociated and plated onto Matrigel-coated 24-well plates at 500,000 neurons per well. At D34, non-targeting siRNA or SETD1A-targeting siRNAs were complexed with Lipofectamine RNAiMAX (ThermoFisher) in Opti-MEM (Gibco) following manufacturer’s instructions and added to the neuronal cultures. For each well, 15 pmol of siRNAs and 3 μl of Lipofectamine RNAiMAX were used. Cells were harvested for RNA 3 days post transfection.

*ChIP-seq data*

ChIP-seq datasets GSM4115890, GSM3508787, GSM4115891 were accessed from Gene Expression Omnibus (GEO) database and visualised using Integrated Genomics Viewer (Broad Institute) using the GRC38/mm10 mouse reference genome.

*ReNVM Immortalised Cell Line Culture*

ReNVM immortalised neural progenitor cells (EMD Millipore SCC008) were maintained in N2B27 media (50% DMEM/F12, 50% Neuro medium, 1% L-Glutamax, 1% MEM Non-Essential Amino Acids supplemented with 1% N2 supplement, and 2% B27 supplement) supplemented with 10ng/ml basic fibroblast growth factor (bFGF, Miltenyi Biotec) and 20ng/ml epidermal growth factor (EGF, Miltenyi Biotec). For neuronal differentiation, ReNVM cells were grown in N2B27 media without growth factor supplementation for 7 days. For astrocyte differentiation, ReNVM cells were grown in B27 media (50% DMEM/F12, 50% Neuro medium, 1% L-Glutamax, 1% MEM Non-Essential Amino Acids supplemented with 2% B27 supplement) with 5% FBS (Gibco) supplemented with 10ng/ml Brain-Derived Neurotrophic Factor (BDNF, Miltenyi Biotec) and 10ng/ml Glial Cell Derived Neurotrophic Factor (GDNF, Miltenyi Biotec) for 7 days.

**Supplementary Tables**

**Supp Table S1: Primers used for CRISPR/Cas-9 genome editing and off-target sequencing**

| **Name** | **Primer sequence (5’ to 3’)** |
| --- | --- |
| SETD1A exon 7 gRNA | TTGAGGATGTGGCACCTACA |
| SETD1A exon 7 sequencing F | GAGAGTGTGCCCTTCGCCCA |
| SETD1A exon 7 sequencing R | TCTGGTTCCCTTCCCTCTCC |
| KAT2A off-target sequencing F | TTTAGTCACCTGGCTCCCCGG |
| KAT2A off-target sequencing R | CAGAAGGAGCCTTACTTGGGGAAGT |
| MACF1 off-target sequencing F | GTGTGGCTATATGTGCTTTGAGACACC |
| MACF1 off-target sequencing R | TCTATTCACCCAAGACACTGCCTTTCT |

**Supp Table S2: Primary antibodies used for immunofluorescence and western blotting**

| **Antibody** | **Catalogue no.** | **Company** | **Dilution** |
| --- | --- | --- | --- |
| Goat anti-Brn2 (C-20) | sc-6029 | Santa Cruz | 1 in 200 |
| Rabbit anti-Cleaved Caspase 3 (Asp175) | 9661 | Cell Signalling Technology | 1 in 200 |
| Rabbit anti-Doublecortin | ab18723 | Abcam | 1 in 500 |
| Rabbit anti-FOXG1 | ab18259 | Abcam | 1 in 300 |
| Rabbit anti-GFAP | ab7260 | Abcam | 1 in 1000 |
| Chicken anti-MAP2 | ab5392 | Abcam | 1 in 1000 |
| Rabbit anti-Nanog | 3580 | Cell Signalling Technology | 1 in 1000 |
| Mouse anti-Nestin (10C2) | ab22035 | Abcam | 1 in 1000 |
| Rabbit anti-Oct3/4 (H-134) | sc-9081 | Santa Cruz | 1 in 1000 |
| Rabbit anti-p21 Waf1/Cip1 (12D1) | 2947 | Cell Signalling Technology | 1 in 1000 |
| Rabbit anti-hSET1 | A300-289A | Bethyl Labs | 1 in 1000 |
| Goat anti-SOX1 | AF3369 | R&D systems | 1 in 200 |
| Mouse anti-SOX2 (E-4) | sc-365823 | Santa Cruz | 1 in 200 |
| Mouse anti-SV2A (E-8) | sc-376234 | Santa Cruz | 1 in 200 |
| Mouse anti-Tubulinβ3 (TUBB3) / TuJ1 | 801202 | Biolegend | 1 in 1000 |
| Mouse anti-α tubulin | Sc-32293 | Santa Cruz | 1 in 500 |

**Supp Table S3: Human primers used for q-RT-PCR**

| **Target** | **Forward primer sequence (5’ to 3’)** | **Reverse primer sequence (5’ to 3’)** |
| --- | --- | --- |
| ACTB | CCAACCGCGAGAAGATGA | CCAGAGGCGTACAGGGATAG |
| BRN2 | GTGCAAGCTGAAGCCTTTGT | CGCTGCGATCTTGTCTATGC |
| FOXG1 | AGGAGGGCGAGAAGAAGAAC | TCACGAAGCACTTGTTGAGG |
| HK1 | CCACCATCTCCACGTTCTTC | TGAGGTTGGACTCATTGTTGG |
| HK2 | AAGCCGTTTCTCCATCTCCT | CTTCTTCACGCAGATCAACC |
| LDHA | GCCAGAGACAATCTTTGGTG | GGCCTGTGCCATCAGTATCT |
| LDHB | GATGGATTTTGGGGGAACAT | AACACCTGCCACATTCACAC |
| NANOG | AATGGTGTGACGCAGAAGGCC | TTGGAAGGTTCCCAGTCGGG |
| PKM | TTCGTCTTTGCAGCGTAGC | ACCGCTCAGAGCTGAATACG |
| POU5F1 | TCAGCCAAACGACCATCTGCC | TTCTCTTTGGGGCCTGCACG |
| SETD1A | TCGAGAGGAAGCTGTGGATACC | CGCCATCTGAGTCAGCATACAG |
| SOX1 | TCCCCCGCCTCAACTG | CAAGGCATTTTGCGTTCACA |
| TUJ1 | GCAGTCGCAGTTTTCACACTC | GGCCAAGGGTCACTACACG |


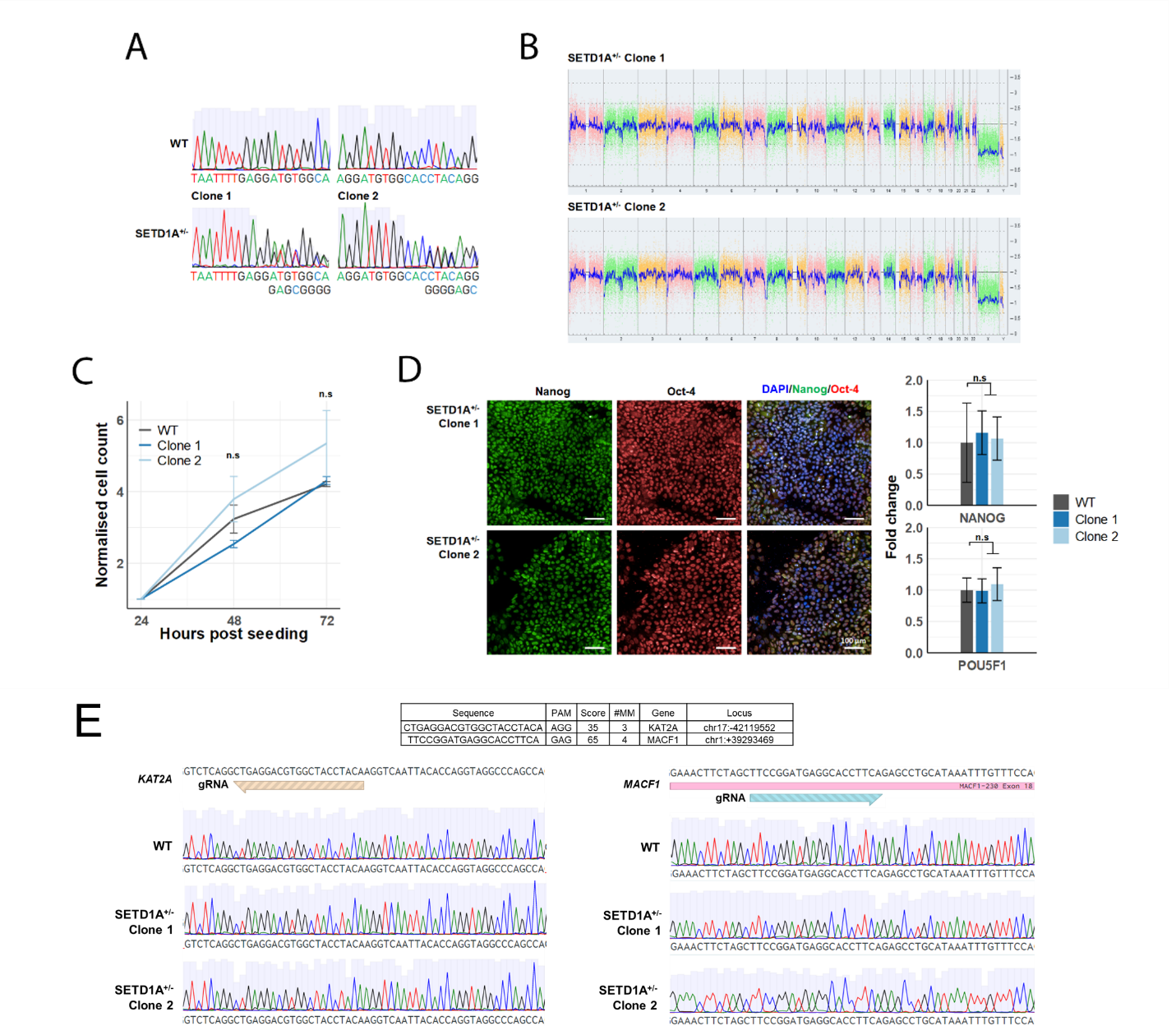


**Supp Figure S1. SETD1A deficient hiPSCs maintain pluripotency and proliferate normally.** A) Representative gDNA sequencing traces of edited locus in SETD1A^+/-^ iPSC lines with WT sequence as a reference. B) Results of KryoStat assay on SETD1A^+/-^ lines showing no detection of chromosomal abnormalities C) Proliferation of WT and SETD1A^+/-^ iPSCs (n=6 wells per line per timepoint, from 3 different passages) D) (left) Representative IF images of pluripotency markers NANOG and Oct-4 (also known as POU5F1) being expressed in SETD1A^+/-^ iPSC clones. (Right) Transcript expression of pluripotency markers in WT and SETD1A^+/-^ iPSC clones (n=3 wells per line, from 3 different passages) E) Table showing off-target sequences and loci with less than 4 mismatches and within coding sequences. (Below) Sanger sequencing traces of respective off-target sites in WT and SETD1A^+/-^ iPSC lines showing no editing at either locus. Data represented are mean ± s.d.


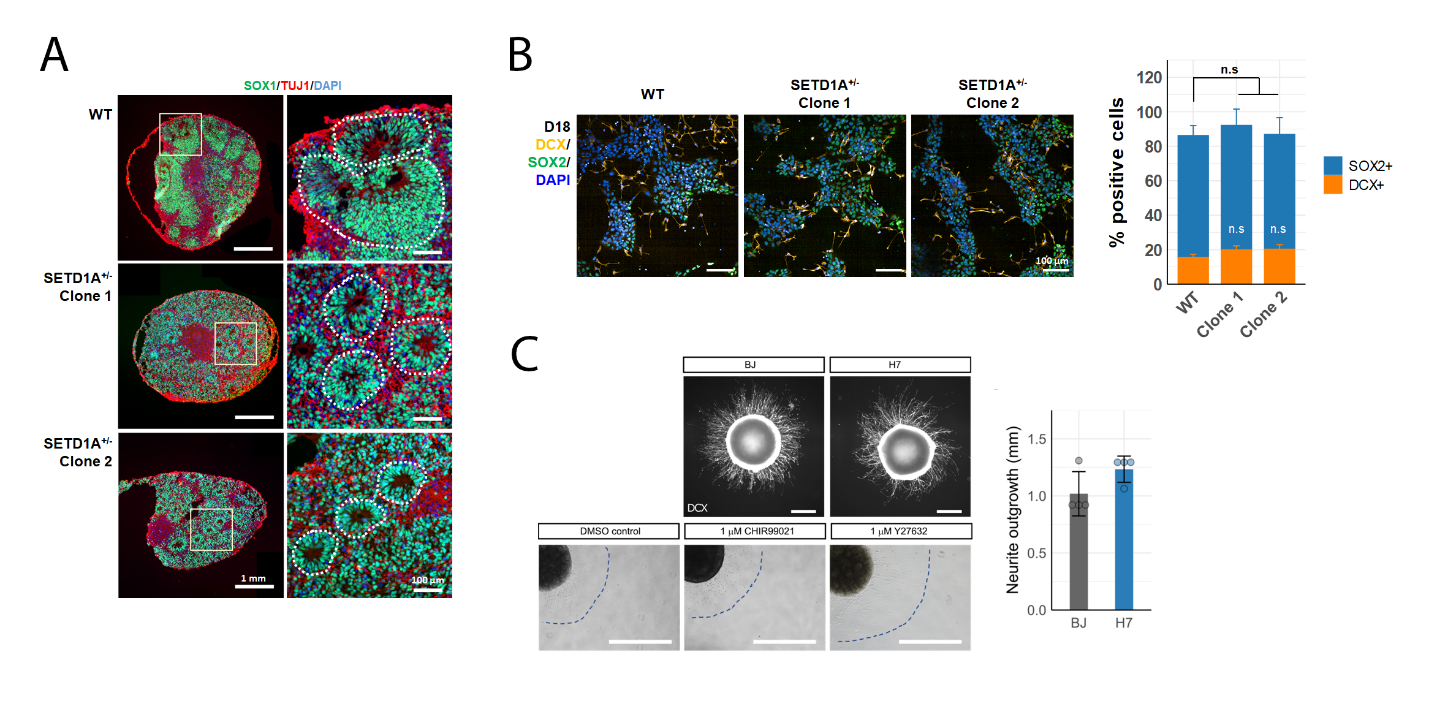


**Supp Figure S2. WT and SETD1A deficient lines differentiate into NPCs and neurons with similar efficiencies.** A) Representative IF images of spheroid sections showing how SOX1^+^ neural rosettes were identified (examples outlined in dashed white line) B) (Left) Representative IF images of SOX2^+^ NPCs and DCX^+^ early neurons obtained from dissociated cortical spheroids at D18 post induction (Right) Percentage of SOX2^+^ NPCs and DCX^+^ neurons at D18 (n = 5 wells per line) C) (Left, top) Representative IF images of WT spheroids (BJ and H7 cell lines) after 48 h of static culture showing DCX^+^ neurite extensions. (Right) Quantification of neurite outgrowth between BJ and H7 spheroids (n=4 spheroids). (Left, bottom) Representative brightfield images of spheroids treated with CHIR99021 and Y27632 with boundary of neurite outgrowth shown with dashed blue line.


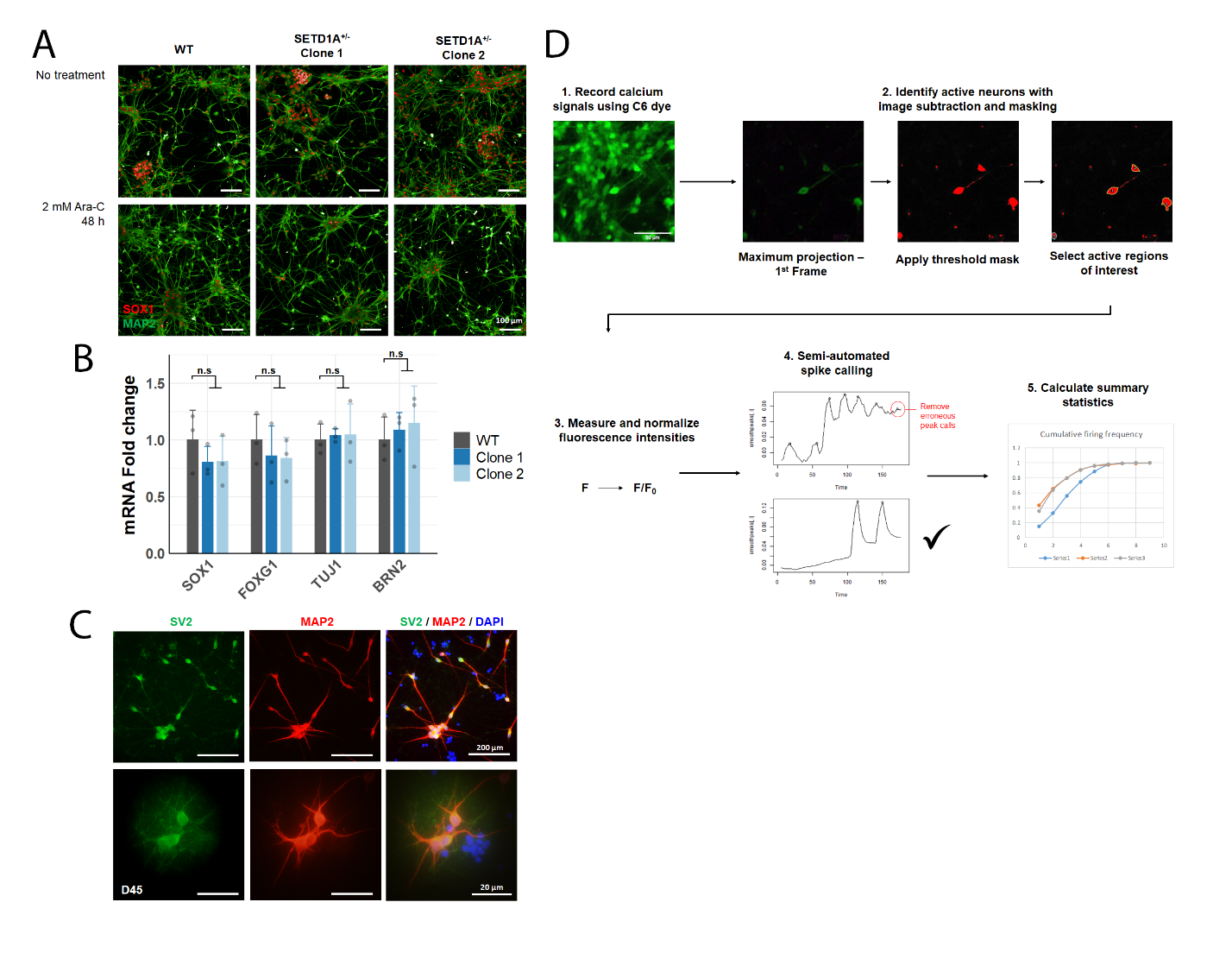


**Supp Figure S3. Derivation of WT and SETD1A^+/-^ neuronal networks for calcium imaging.** A) Representative IF images showing presence of SOX1^+^ NPCs in neuronal cultures without Ara-C treatment and enrichment for MAP2^+^ neurons after 48 h of Ara-C. B) Transcript level expression of NPC (SOX1, FOXG1) and neuron (TUJ1, BRN2) markers in neuronal networks at D45 post induction (n = 3 wells per line from 3 independent differentiations) C) Representative IF images of expression of synaptic marker SV2 in MAP2^+^ WT neurons at D45 post-induction D) Schematic of calcium imaging analysis workflow showing identification of active neurons by image calculations and spike identification using the peakPick package in R. Data represented are mean ± s.d. For all comparisons, one-way ANOVA with Dunnett’s multiple comparisons test was performed.


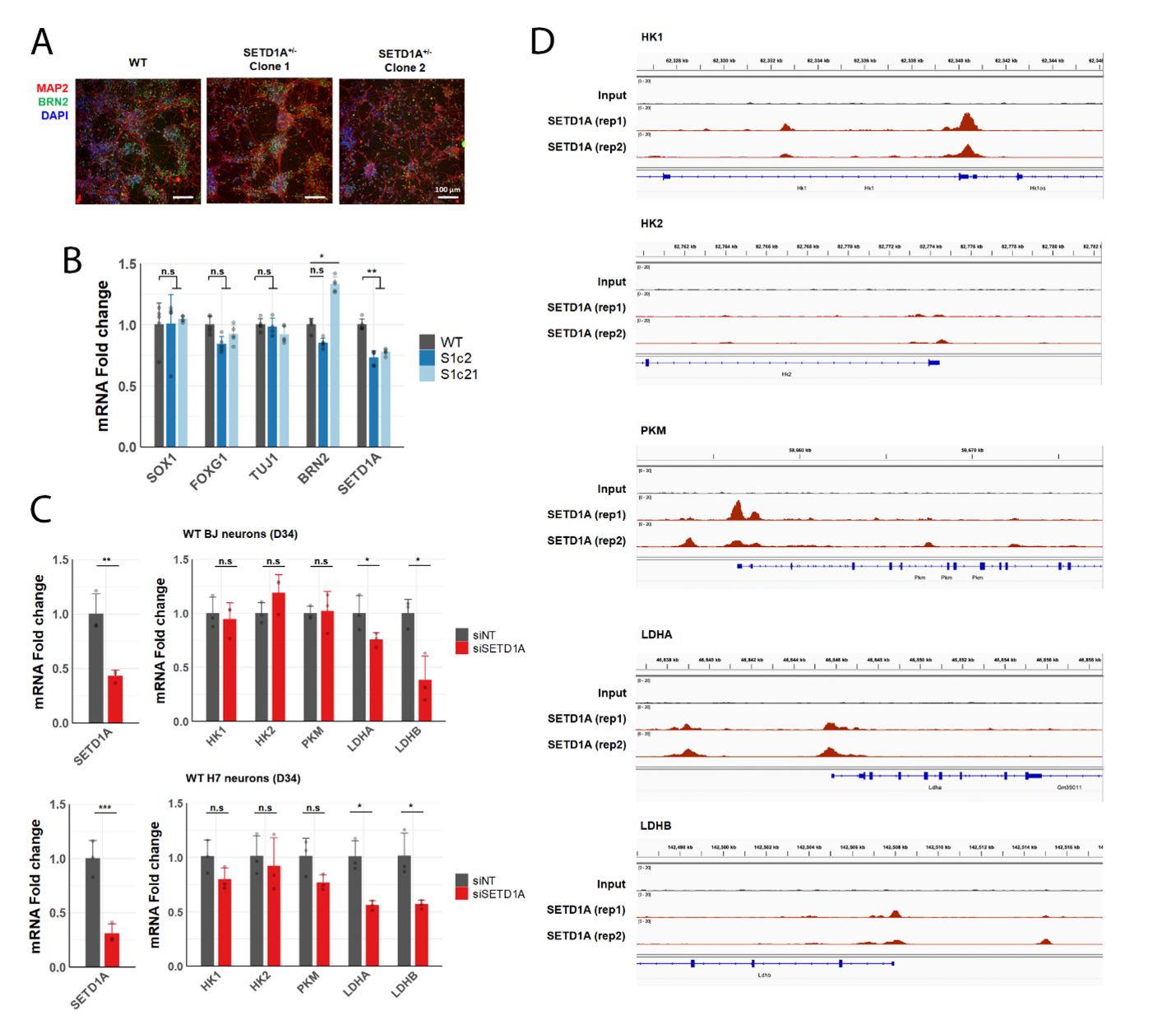


**Supp Figure S4. SETD1A modulates expression of glycolytic genes in neurons.** A) Representative IF images of BRN2^+^/MAP2^+^ neurons at D34 post-induction B) Transcript level expression of NPC (SOX1, FOXG1) and neuron (TUJ1, BRN2) markers, and SETD1A in cortical spheroids at D30 post-induction as measured by qRT-PCR (n=5 batches per line, from 3 independent differentiations) C) Transcript level expression of SETD1A and glycolytic enzymes in response to SETD1A knockdown using siRNA in WT neurons derived from BJ and H7 cell lines at D34 post-induction (n=3 wells from 3 independent differentiations) D) SETD1A ChIP-seq dataset of mouse prefrontal cortex (GSM4115890, GSM3508787, GSM4115891) from Gene Expression Omnibus (GEO) database indicates possible binding of SETD1A to the promoter regions of HK1, PKM and LDHA in neurons. Data represented are mean ± s.d. For all comparisons, one-way ANOVA with Dunnett’s multiple comparisons test was performed. * p<0.05; ** p<0.01; *** p<0.001.


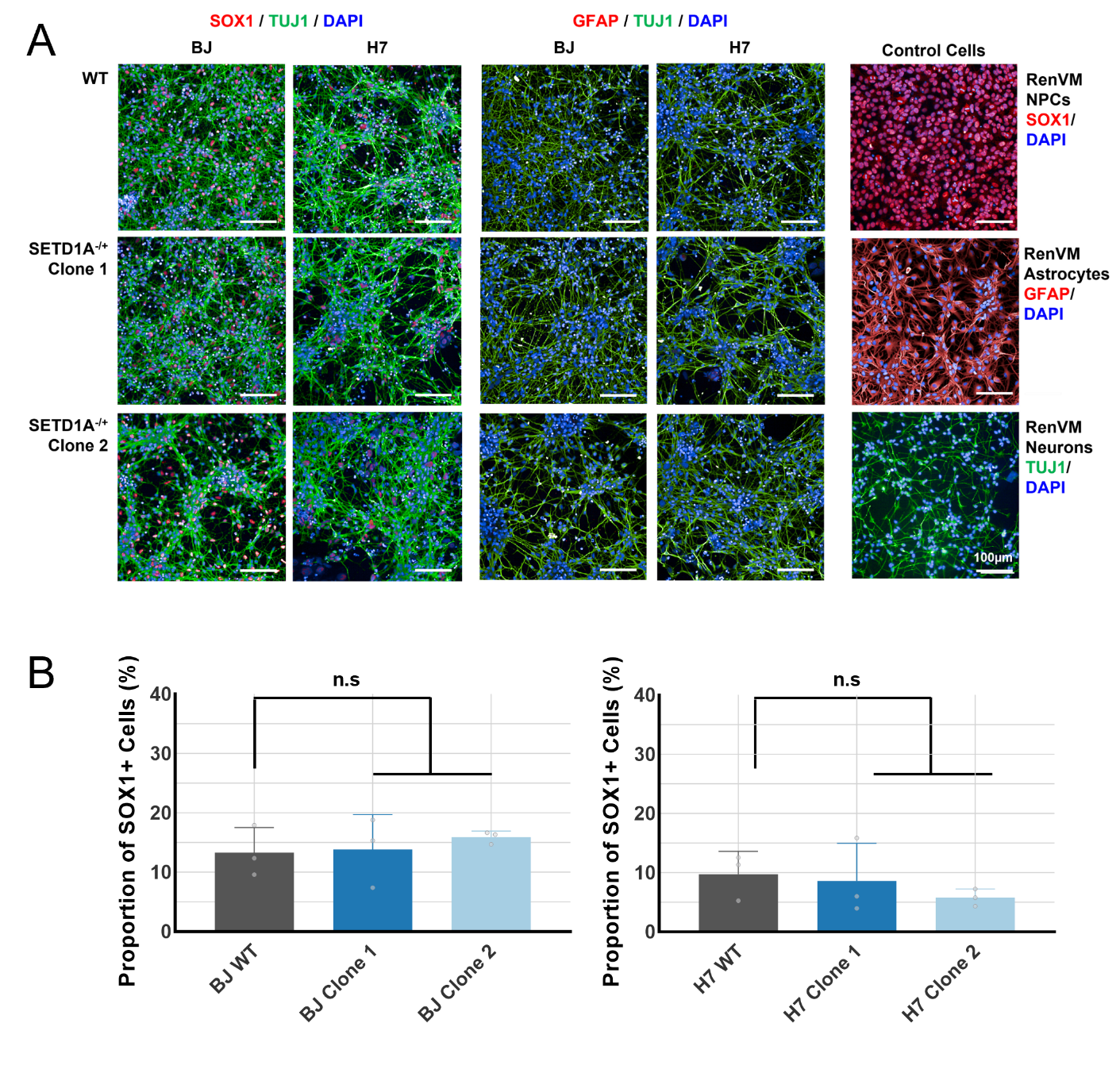
**Supp Figure S5. BJ iPSC and H7 ESC WT and SETD1A lines showed similar composition of NPCs**A) Representative IF Images showing the presence of SOX1+ NPCs and an absence of GFAP+ astrocytes for D34 cultures that were seeded for the seahorse XF experiments. Control ReNVM immortal cell lines were included as staining controls for SOX1+ and GFAP+ staining. B) Quantification of SOX1+ staining for BJ SETD1A clones and H7 SETD1A clones, 3 wells per line. Data represented are mean ± s.d unless otherwise. For all comparisons, one-way ANOVA with Dunnett’s multiple comparisons test was performed.


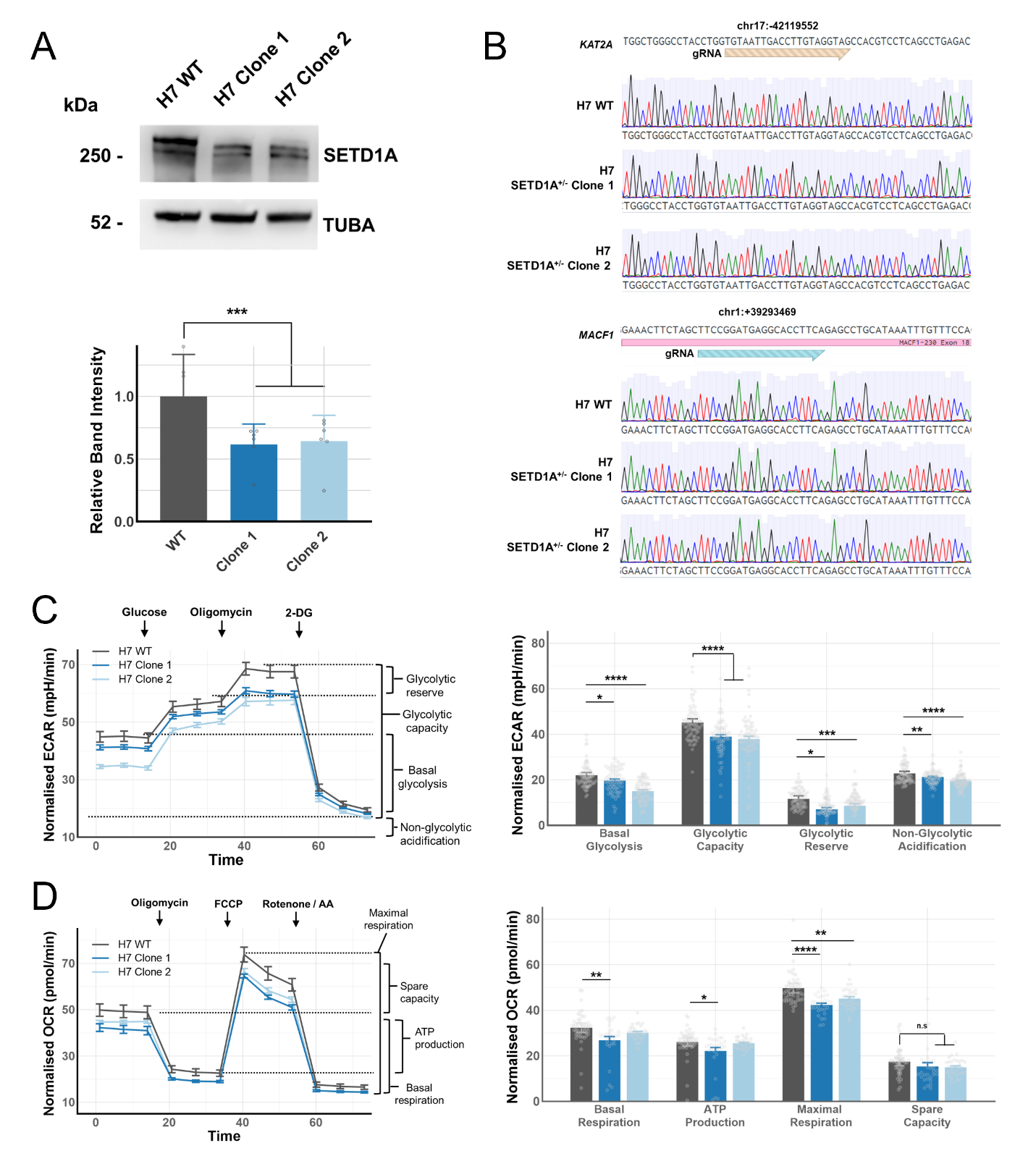
**Supp Figure S6 H7 SETD1A^+/-^ exhibit similar metabolic defects in glycolysis and mitochondrial respiration** A) Representative bands from western blot of SETD1A in H7 ESC and SETD1A^+/-^ H7 ESC and relative quantification of band intensity (n = 6 wells from 2 independent passages). B) Sanger sequencing traces of respective off-target sites in WT H7 ESC and SETD1A+/- H7 ESC lines showing no editing at either locus. C) Results of glycolytic stress assay in WT H7 and H7 SETD1A^+/-^ neurons at D34. (left) Seahorse curves showing basal glycolysis and responses to 10 mM glucose, 2 μM oligomycin and 50 mM 2-DG over time (mins). Extracellular acidification rate (ECAR) was normalised to nuclei number post assay. (right) Quantification of glycolytic function from the same experiments (error bars = s.e.m, n = 76 well (H7 WT), 80 wells (H7 SETD1A^+/-^ Clone 1), 74 wells (H7 SETD1A^+/-^ Clone 2) from 3 independent differentiations). E) Results of mitochondrial stress assay in H7 WT and H7 SETD1A^+/-^ neurons at D34. (left) Seahorse curves showing basal respiration and responses to 2 μM oligomycin, 1 μM FCCP, and 0.5 μM rotenone / antimycin-A over time (mins). Oxygen consumption rate (OCR) was normalised to nuclei number post assay. (right) Quantification of respiratory function from the same experiments (error bars = s.e.m, n = 46 wells (H7 WT), 30 wells (H7 SETD1A^+/-^ Clone 1), 32 wells (H7 SETD1A^+/-^ Clone 2) from 3 independent differentiations). Data represented are mean ± s.d unless otherwise. For all comparisons, one-way ANOVA with Dunnett’s multiple comparisons test was performed. * p<0.05; ** p<0.01; *** p<0.001; **** p<0.0001.
